# Lexical and Grammatical Aspect in On-line Processing of English Past Tense and Progressive Aspect by Mandarin Speakers

**DOI:** 10.3389/fpsyg.2021.661923

**Published:** 2021-06-10

**Authors:** Xiaoyan Zeng, Xiaoxiang Chen, Yasuhiro Shirai

**Affiliations:** ^1^School of Foreign Languages, Hunan University, Changsha, China; ^2^Department of Cognitive Science, Case Western Reserve University, Cleveland, OH, United States

**Keywords:** lexical aspect, grammatical aspect, English past tense, progressive aspect, tense-aspect processing, Mandarin speakers, foreign language learning

## Abstract

Previous studies have shown that the grammatical aspect of verb predicates has an effect on tense-aspect sentence processing. However, it remains unclear as to whether the interaction of lexical aspect and grammatical aspect can influence the form-meaning association in the second language (L2) tense-aspect sentence processing, especially for the learners whose native language is grammatically marked differently from their L2. This study conducts a psycholinguistic investigation to highlight how the prototypical and non-prototypical associations predicted in the Aspect Hypothesis and L2 proficiency level influence the processing of English past tense and progressive morphology by Mandarin Chinese learners at two proficiency levels and native English speakers. The results show that the prototypical associations of English tense-aspect categories predicted in the Aspect Hypothesis, such as achievement verbs with past tense and activity verbs with the progressive aspect, can engender shorter reading time than non-prototypical associations for both native speakers and second language learners. There is no significant difference between native speakers and Chinese learners of English in their processing of prototypical items, while significant differences exist in the processing of non-prototypical items. The L2 proficiency level does not have an effect on the processing of prototypes but on the processing of non-prototypes in the L2 tense-aspect marking. This study extends previous research, showing the interaction effect of lexical aspect and grammatical aspect in the form-meaning association in L2 tense-aspect sentence processing.

## Introduction

In the studies of L2 acquisition of tense-aspect morphology, the prototypical associations, such as achievement verbs, with past tense and activity verbs with the progressive aspect, have been observed and summarized in the Aspect Hypothesis (Shirai, 1991; Andersen and Shirai, 1994; Bardovi-Harlig, 2000; Bardovi-Harlig and Comajoan-Colomé, 2020). It has been suggested that this is because of the compatibility of the semantic representation of lexical and grammatical aspect. That is, the combinations of telic verbs with the perfective aspect, and activities with progressive marking are more compatible, natural, prototypical, and frequent in language use. The frequency distribution and the cognitive-based prototype account have been empirically supported from a large number of offline studies (e.g., Li and Shirai, 2000) and corpus-based studies (Fuchs and Werner, 2018). However, even if explicit knowledge of a structure might have been acquired, possibly *via* classroom instructions, learners may not be able to make use of this knowledge in real-time processing (Van Patten et al., 2012).

Moreover, the mechanism of how these form-meaning associations emerge is still not well understood. Whether the aspectual knowledge that learners display in the off-line tasks can be applied automatically in online comprehension tasks is still unclear. Research on L2 acquisition of English tense and aspect has not been conducted as widely from a language processing perspective as it has from a production perspective. More importantly, the frequency distribution effect has not been well-recognized in the area of language processing of tense-aspect markers. Most previous processing studies have focused on the effect of grammatical aspect. The interactive effect of lexical aspect and grammatical aspect has been under-explored in the L2 learners’ tense-aspect processing. This study investigated the effects of lexical aspect and the L2 proficiency level on the processing of English past tense and progressive morphology by exploring the Chinese learners at two L2 proficiency levels of the native English speakers.

Here, we briefly define technical terms crucial to understanding the linguistic phenomenon under investigation. Linguists distinguish grammatical aspect from lexical aspect. Grammatical aspect, often referred to as “viewpoint aspect,” (e.g., Smith, 1997) grammatically encodes how a speaker views a situation – whether it is viewed as a whole (the perfective aspect, e.g., *He walked to the store*) or as having an internal structure (the imperfective aspect, e.g., *He was walking to the store*). Lexical aspect concerns temporal semantics of verbal predicates [most commonly used is Vendler’s (1957) four-way classification (states, activities, accomplishments, and achievements)]. States encode a situation as homogeneous, with no end points or successive phrases or dynamicity (e.g., *know and love*). Activities characterize a situation as having successive phases over time with no inherent end point (e.g., *run* and *walk*). Accomplishments encode a situation as consisting of having successive phrases (e.g., *build a house*) with an inherent end point, after which the situation cannot continue. Achievements encode a situation as punctual and instantaneous, having no duration (e.g., *fall* and *reach the summit*). Accomplishments and achievements are telic (involving a natural end point) while states and activities are atelic (no natural end point; Vendler, 1957; Li and Shirai, 2000).

## Literature Review

The acquisition studies on tense-aspect marking facilitate the understanding of the mechanism behind the form-meaning association in language acquisition (Sugaya and Shirai, 2007). In both L1 and L2 acquisition, learners are observed to be sensitive to the inherent lexical aspect of verbs in acquiring tense-aspect morphology. The Aspect Hypothesis (henceforth, AH, Andersen and Shirai, 1996; Bardovi-Harlig, 2000) comprises four generalizations about learners’ acquisition of tense-aspect marking:

1. Learners first use past marking (e.g., English) or perfective marking (Chinese, Spanish, etc.) on achievement and accomplishment verbs, eventually extending its use to activities and stative verbs.
2. In languages that encode the perfective/imperfective distinction, imperfective past appears later than perfective past, and imperfective past marking begins with stative verbs and activity verbs, then extending to accomplishment and achievement verbs.
3. In languages that have a progressive aspect, progressive marking begins with activity verbs, then extends to accomplishment or achievement verbs.
4. Progressive markings are not incorrectly overextended to stative verbs (Andersen and Shirai, 1996, p. 533; see also Shirai, 1991, p. 9–10)

These generalizations are schematically summarized in Table 1 below.

**Table 1:** Predicted order of development of tense-aspect morphology (adapted from Li and Shirai, 2000, p. 50).

	State		Activity		Accomplishment		Achievement
Perfective past	4	<=====	3	<=====	2	<=====	1
Imperfective past	1	=====>	2	=====>	3	=====>	4
Progressive	?	<=====	1	<=====	2	<=====	3

? means combination rarely occurs.

The AH thus predicts that learners are strongly influenced by verbal semantics in acquiring tense-aspect markers. That is, past perfective markers are associated with telic verbs (achievements and accomplishments, with achievements as the prototype), while general imperfective markers are associated with atelic verbs (activities and states, with states as the prototype), and progressive markers (i.e., dynamic imperfective) with activity verbs as the prototype. Shirai and Andersen (1995) explain this by proposing that activity verbs, which are dynamic, durative, and atelic, exemplify the most typical combinations for the progressive marking (i.e., prototypical progressive), while achievement verbs, which are punctual and telic, exemplify the most typical connections with past tense morphology (i.e., prototypical past). The evidence for the association of the perfective aspect with telics and the progressive with activity verbs is robust in offline acquisition studies in the literature (e.g., Salaberry, 1999; Bardovi-Harlig, 2000; Shirai, 2002).

To explain these observations, Andersen and Shirai (1994, 1996) proposed the Prototype Hypothesis, which states that language learners initially acquire the prototypes for each aspectual marking (i.e., perfective/past morphology with accomplishment/achievement verbs, and progressive morphology with activity verbs), and then gradually extend their scope to less-prototypical exemplars. They argued that the learners at the beginning stage are restricted to the prototypes of the linguistic category, and then only later can they freely apply those markers to more peripheral members. This observation shows a close relationship between learners’ use of tense-aspect marking and temporal semantics of verbal predicates. However, further investigation is needed to explore whether L2 prototype knowledge formation has an effect underlying the identification of aspectual values for grammatical morphemes in the L2 learners’ tense-aspect processing.

Most previous studies on tense-aspect processing employed an agreement violation paradigm and/or a self-paced reading technique to investigate whether the knowledge of tense-aspect marking that learners displayed in offline performance tasks could be applied automatically to their online comprehension. For example, Roberts and Liszka (2013) used an offline acceptability judgment and a self-paced reading experiment to measure L2 English learners’ explicit and implicit knowledge about their sensitivity to tense-aspect mismatches between a temporal adverbial and an inflected verb (i.e., a mismatch between past adverbial and the present perfect form in English, such as *He has arrived last week*, which is ungrammatical in English but not in the learners’ L1). The results indicate that all participants demonstrated explicit knowledge about the offline task while the self-paced reading task showed that there is a crosslinguistic influence during online L2 processing; that is, whether learners’ first language encodes the aspect grammatically or not was important in that French speakers, whose L1 encodes aspect grammatically was sensitive to the mismatch, but not German learners (more on this study below).

Previous reading time studies revealed that native speakers exploit grammatical aspectual cues such as perfective and imperfective morphology when constructing mental situation models (e.g., Carreiras et al., 1997; Ferretti et al., 2007). Some studies, which examined the effect of grammatical aspect on sentence processing, found that sentences with perfective aspect are often processed more quickly than imperfective ones (e.g., Madden and Zwaan, 2003 in English; Yap et al., 2006 in Japanese). For example, Madden and Zwaan (2003) used three picture-sentence matching tasks and found that native English participants matched perfective sentences with pictures depicting completed situations more quickly than with pictures depicting ongoing situations. They commented that the perfective facilitation effect is attributed to the perfective-imperfective contrast in the grammatical marking of the aspect. However, this study used only accomplishment verbs, i.e., verbs with an inherent end point.

Recently, the interaction of lexical and grammatical aspect has drawn more attention in the area of language processing. Yap et al. (2009) investigated native Cantonese speakers’ reaction times (RTs) to explore the effects of both lexical aspect and grammatical aspect on the language processing in Cantonese. Employing auditory processing, they manipulated the combinations of lexical and grammatical aspect (i.e., accomplishment with perfective aspect *zo* and activity with imperfective aspect *gan*) and tested whether they would yield faster cognitive processing than less semantically compatible combinations. The results showed a strong prototype effect in the interaction between lexical aspect and grammatical aspect. That is, perfective sentences were processed more quickly with accomplishment verbs, while imperfective sentences were processed more quickly with activity verbs relative to other conditions. This study provided the most compelling evidence for a prototype account for aspectual processing within native Cantonese speakers. Yap et al. (2009) argued that prototypical associations of tense-aspect categories would engender shorter reading times. They provided a basic cognitive principle, namely semantic compatibility to account for their findings. The semantic compatibility exists in the association between accomplishment verbs and the bounded features of the perfective aspect and between activity verbs, and the unbounded features of the imperfective aspect.

However, the issue of whether there is a prototype effect on tense-aspect processing is still controversial. Unlike Yap et al. (2009), which provided strong support to a prototype representation of tense-aspect categories within native Cantonese speakers, Chan (2012) did not find such online processing biases for L2 learners of English. Using a self-paced reading task, Chan (2012) undertook a psycholinguistic investigation into native English speakers’ and English L2 learners’ processing of English past and progressive morphology. Three types of lexical aspect (state, activity, and achievement) and two grammaticized tense-aspect categories (past tense and progressive aspect) were investigated. The results showed that L2 learners did not have uniform processing advantages afforded by tense-aspect prototypes. The Korean participants followed the PAST prototype, while the German participants did not. Both the German and Chinese participants processed state PAST the quickest, which does not support the prediction of the prototype hypothesis. For the PROG prototype, no evidence was presented from any L1 groups. However, the data from the native English speakers provided support for PAST and PROG prototypes, although the reading time trends observed were not statistically significant. Therefore, further empirical studies are necessary to validate the prototype account and explore its processing consequence among L2 learners.

The L2 processing of tense-aspect morphology often focuses on the difference between L1–L2 pairings and participants of different proficiency levels. The issue of whether L2 tense-aspect processing is influenced by aspectual features in learners’ native language is still open. Some researchers argued that grammaticized aspectual categories in L1 (e.g., the lack of progressive aspect in German) have a vital impact on ultimate L2 attainment, especially regarding the principles of event construal in language production (e.g., von Stutterheim and Carroll, 2006). It was found in von Stutterheim and Nüse (2003) that, in the retelling of a silent film and verbalization of short video clips, German speakers tend to infer temporal situations more holistically than English speakers. Similar results have also been found in experiments, looking at L2 language production and processing (e.g., von Stutterheim and Carroll, 2006). In the study, von Stutterheim and Carroll (2006) asked both advanced German learners of English and advanced English learners of German to orally describe the situations they had watched in short film clips. Then, their speech was transcribed and coded based on whether an end point was explicitly mentioned. Results showed that the German learners of English reported end points at a higher rate than the English learners of German (36.7 vs. 31.6%). What is more, the native German speakers mentioned end points in German significantly more frequently than the native English speakers did in English (76.4 vs. 25.2%). The authors explained that the native German speakers as well as the German learners of English extended a general tendency to conceptualize a situation holistically; therefore, they linguistically encode and report the end points. While, for English speakers, the English progressive, in contrast, is a highly automatized grammatical option that enables them to report a situation in any intermediate phase before culmination. This study showed L2 tense-aspect processing is somewhat influenced by learners’ L1. Findings from von Stutterheim et al. (2012) further showed, in an eye-tracking study utilizing short videoclips, that native speakers with progressive marking (e.g., English) pay more attention to the process leading to the end point than native speakers of languages without progressive marking (e.g., German), who tend to pay more attention to the end point. The findings of von Stutterheim and associates can be explained with Slobin’s (1996) claim – “thinking for speaking,” which posits that “one fits one’s thoughts into available linguistic forms” (Slobin, 1987; as cited in Slobin, 2003).

Using self-paced reading experiments, Roberts and Liszka (2013) investigated the role of L1 in real-time processing of L2 tense-aspect morphology among advanced French and German learners of English as an L2 to see if they are sensitive to tense-aspect mismatches between a fronted temporal adverbial (e.g., yesterday) and the inflected verb that follows (e.g., present perfect). Results showed that only the French L2 learners, whose L1 has grammaticized aspect, were sensitive to the mismatched conditions in both the present perfect contexts and the past simple; whereas the German L2 learners did not show a processing cost at all for either the mismatched type or matched one. The authors explained that the differences in performance between the L2 groups come from the learners’ native language. This study concluded that, in L2 tense-aspect processing, only learners whose L1 has grammaticized aspect were sensitive to the tense aspect violations online; thus, the L2 tense-aspect processing is influenced by aspectual features in learners’ L1. Roberts and Liszka (2019) also found out the L1 effect on L2 processing and offline interpretations of aspectual distinction. The authors argued that whether a learner’s native language encodes progressive aspect *via* syntactic or only lexical means influences his/her interpretations of aspectual distinction.

Chan (2012) investigated what is universal and what is language specific about L2 tense-aspect processing. The participants in this study included native English speakers as well as English L2 learners of L1 German, Korean, and Mandarin Chinese, which differ systematically in terms of past and progressive morphology. L1 effects were found not only in prototypes in processing L2 tense-aspect distinction but also in processing consequences of the non-prototypical combination of grammatical aspect and lexical aspect (e.g., *the kid was jumping into the swimming pool*; achievement predicate and progressive marking) in L2 learners.

According to the predictions of the Aspect Hypothesis, learners’ dependence on lexical aspect decreases as the proficiency level increases, as noted earlier. Some production studies tested this prediction; however, findings in the production experiments show the effect of lexical aspect decreases as the proficiency level goes up (e.g., Rocca, 2007) while others show this effect increases (Robison, 1995a).

Regarding proficiency effect on L2 tense-aspect processing, an interesting question is whether advanced learners can perform as successfully as native speakers (i.e., ultimate attainment). Von Stutterheim and her colleagues conducted a series of psycholinguistic studies on bilingual speakers’ representation and linguistic encoding of events. Their findings showed that very advanced L2 learners succeeded in using their target languages correctly but failed to show native-like performance on a number of measurements (von Stutterheim and Nüse, 2003; von Stutterheim and Carroll, 2006).

The current study focuses on how the prototypical and non-prototypical associations predicted in the Aspect Hypothesis and the L2 proficiency level influence the processing of English past tense and progressive morphology by investigating two proficiency levels of Mandarin Chinese EFL learners and native English speakers as a control group. Two research questions will be addressed below:

1. How does the lexical aspect of verbs influence L2 learners’ and native speakers’ sentence processing, respectively? Specifically, how fast are activity verbs, achievement verbs, and states processed by L2 learners and native speakers in simple past tense and present progressive?
2. How does the learners’ L2 proficiency level influence their processing of L2 English tense-aspect marking? Will they perform in the same way as native speakers?

## Materials and Methods

### Participants

The learner participants in this study were recruited from two universities in Central South China. They completed a language history questionnaire and only those who had no study experience in English-speaking countries were included in this experiment. Thirty non-English majors who have passed CET-4 (College English Test Band 4) but with scores lower than 450, and thirty English majors who have passed TEM-8 (Test for English Majors Band 8) with scores higher than 80 were chosen as the participants for this study. All these participants completed a standardized English proficiency test, namely, the Quick Placement Test (QPT) by Oxford University Press. QPT consists of 60 multiple-choice items on grammar, vocabulary, and reading, with a maximum score of 60. Thirty non-English majors (16 women, 14 men, mean age: 19.6 years, age range: 18–22 years) and 30 English majors (18 women, 12 men, mean age: 20.2 years, age range: 18–23 years) participated in the experiment.

English native participants were recruited from an American university. Thirty native speakers (16 women, 14 men, mean age: 20.5 years, age range: 18–22 years) who had no study experience in non-English-speaking countries were grouped into the native English speaker group (NS for brevity).

All the participants were right-handed with normal vision or corrected-to-normal vision. All the participants in the study were compensated for their participation. Participant profile information, the results of the Quick Placement Test (QPT), and the questionnaire are given in Table 2.

**Table 2:** Participant profile information.

	NS (*N* = 30)		CH_L (*N* = 30)		CH_H (*N* = 30)	
	*M*	*SD*	*M*	*SD*	*M*	*SD*
Age	20.5	0.67	19.6	1.35	20.2	1.56
Self-rating						
Speaking	na	na	4.43	1.1	4.87	0.86
Listening	na	na	4.97	0.93	5.53	0.68
Reading	na	na	5.2	0.85	5.67	0.48
Writing	na	na	4.03	1.03	5.47	0.68
Quick placement Test	na	na	45.47	6.14	50.13	6.02
Beginning age of English instruction	na	na	6.97	1.13	7.03	1.19

There is no significant difference between the English majors (*M* = 7.03, *SE* = 0.217) and non-English majors (*M* = 6.97, *SE* = 0.206) in their beginning age of English instruction, *p* = 0.825. The English majors were significantly more proficient in English (*M* = 50.13, *SE* = 1.1) than non-English majors (*M* = 45.47, *SE* = 1.12), *p* = 0.004 in the Quick Placement Test. This result was also in line with the participants’ self-ratings of their speaking, listening, reading, and writing skills on a seven-point scale. Seven indicates native-like proficiency. The results of the independent-samples *t*-tests confirmed that the scores of the four skills of the non-English majors were much lower than those of the English majors (all *ps* < 0.05). Therefore, the less-proficient non-English major participants were grouped into the Chinese learners with a lower-English-proficiency level (CH_L). The 30 English major participants were grouped as Chinese learners with a higher-English-proficiency level (CH_H).

### Stimuli

The stimuli in this experiment consisted of 144 sentences marked for English simple past tense and present progressive aspect in triplets. The critical verbs of each of the triplets vary across three lexical aspect classes: state, activity, and achievement. The classification of verb predicates is based on the tests used in Shirai and Andersen (1995), both in Chan (2012) and in the current study. Table 3 presents sample stimuli sentences in this study. The complete list of target stimuli is in the Supplementary Material.

**Table 3:** Sample stimuli.

Lexical aspect	Grammatical tense and aspect	
	PAST	PROGRESSIVE
State	Bill *loved the innocent* child in the *playground*.	Tom is *hoping to win* the game on *Saturday*.
Activity	Bill *helped the innocent* child in the *playground*.	Tom is *training to win* the game on *Saturday*. (Prototype progressive)
Achievement	Bill *killed the innocent* child in the *playground*. (Prototype past)	Tom is *beginning to win* the game on *Saturday*.

In the present study, since we did not test accomplishments, often considered to be a somewhat intermediate category among Vendler’s four classes (e.g., Jacobsen, 1992), all combinations other than the prototypes are considered non-prototypes. Namely, for the progressive aspect, activities are prototypical while both states and achievements are non-prototypical, and, for the past tense, achievements are the prototype while states and activities are non-prototypical (see Table 3).

The critical verbs are underlined. The italic words highlight the word regions where reading times in these regions were analyzed. The stimuli were adapted from Chan (2012), except that (1) we only used regular verbs for past tense items, and (2) we also took the number of orthographic neighborhood density into consideration, which Chan (2012) did not. Following Chan (2012), this study adopted subjects of the sentences to be constructed as general as possible to offset any anticipatory priming effects during comprehension. All critical verbs marked with English past tense marking and progressive marking were checked for token frequencies according to the Corpus of Contemporary American English (COCA), whose token counts are based on a corpus of 560 million words by searching specific verbs marked with pos tags of past or progressive inflection. For example, for the verb “attended,” search “[attend]_VVD” or for the word “finding,” search “[find]_VVG.” [VVD] means verbs with the past tense, and [VVG] means -ing participle of lexical verb. Table 4 shows the characteristics of the stimuli. Specifically, the mean log token frequencies of the critical verbs with past tense marking and those with progressive marking were obtained from COCA. Following Chan (2012), this study excluded verb participle, gerund, adjective, and noun counterparts that share identical forms with the target verbs in token frequency counts. The number of orthographic neighbors of the verbs (neighborhood density), defined as “the number of other words of the same length that share all but one letter in the same position” (Grainger et al., 2005), has also been taken into consideration because it has been shown to affect visual word recognition (Frost et al., 2000). The orthographic neighborhood density data were extracted, using the English Lexicon Project Database (Balota et al., 2007; see Supplementary Table 1 and Supplementary Figure 1). To ensure that the critical verbs were of comparable properties, one-way ANOVAs for length, frequency, and neighborhood density were conducted.

**Table 4:** Mean (SD) length, frequency, and orthographic neighborhood density of critical verbs in their inflected forms.

	State		Activity		Achievement	
	*M*	*SD*	*M*	*SD*	*M*	*SD*
PAST						
Length	6.3	1.49	7.2	1.69	7.1	1.2
Frequency	4.24	0.6	3.94	0.49	4.08	0.62
Orthographic neighborhood	2.6	1.9	2.5	2.6	3.8	3.08
PROG						
Length	7.4	1.51	7.4	0.52	7.5	1.43
Frequency	4.04	0.66	4.34	0.43	4.51	0.72
Orthographic neighborhood	2.6	2.55	2.5	1.72	3	3.09

As is seen in Table 4, there was no significant difference in word length across lexical aspect in PAST, *F* (2, 27) = 1.121, *p* = 0.341. No significant difference regarding word length in PROG across lexical aspect, *F* (2, 27) = 0.022, *p* = 0.978 was found. There was no significant difference in word length across lexical aspect classes in PAST and PROG, *F* (5, 54) = 1.054, *p* = 0.396. Similarly, there was no significant difference in word frequency across lexical aspect in PAST, *F* (2, 27) = 0.687, *p* = 0.512. No significant difference regarding word frequency in PROG across lexical aspect, *F* (2, 27) = 1.477, *p* = 0.246 was found. There was no significant difference in word frequency across the lexical aspect in PAST and PROG, *F* (5, 54) = 1.272, *p* = 0.289. The analysis showed no significant differences in terms of orthographic neighborhood density in PAST and PROG; *F* (2, 27) = 0.792, *p* = 0.463 and *F* (2, 27) = 0.111, *p* = 0.896, respectively.

The stimuli were distributed over three versions in triplets, using the Latin Square design. Each version contained 24 different sentences – eight for each of the three conditions (state, activity, and achievement) for both the past tense and the progressive marking. All sentences just appeared only once in any of the versions, and the participants only saw one sentence from any given triplets. Altogether, 144 filler sentences (48 for each participant) were presented randomly to prevent the participants from developing inferring and guessing strategies for reading the stimuli. These filler sentences were unrelated to the experiments in this study, and they were obtained from Schwartz and Kroll (2006) and Chan (2012). To prevent the participants from pressing the spacebar mechanically and to ensure meaningful reading comprehension, a yes/no comprehension question prompt was presented with each of the filler sentences embedded throughout the experiment.

### Experimental Procedure

**Figure 1:**
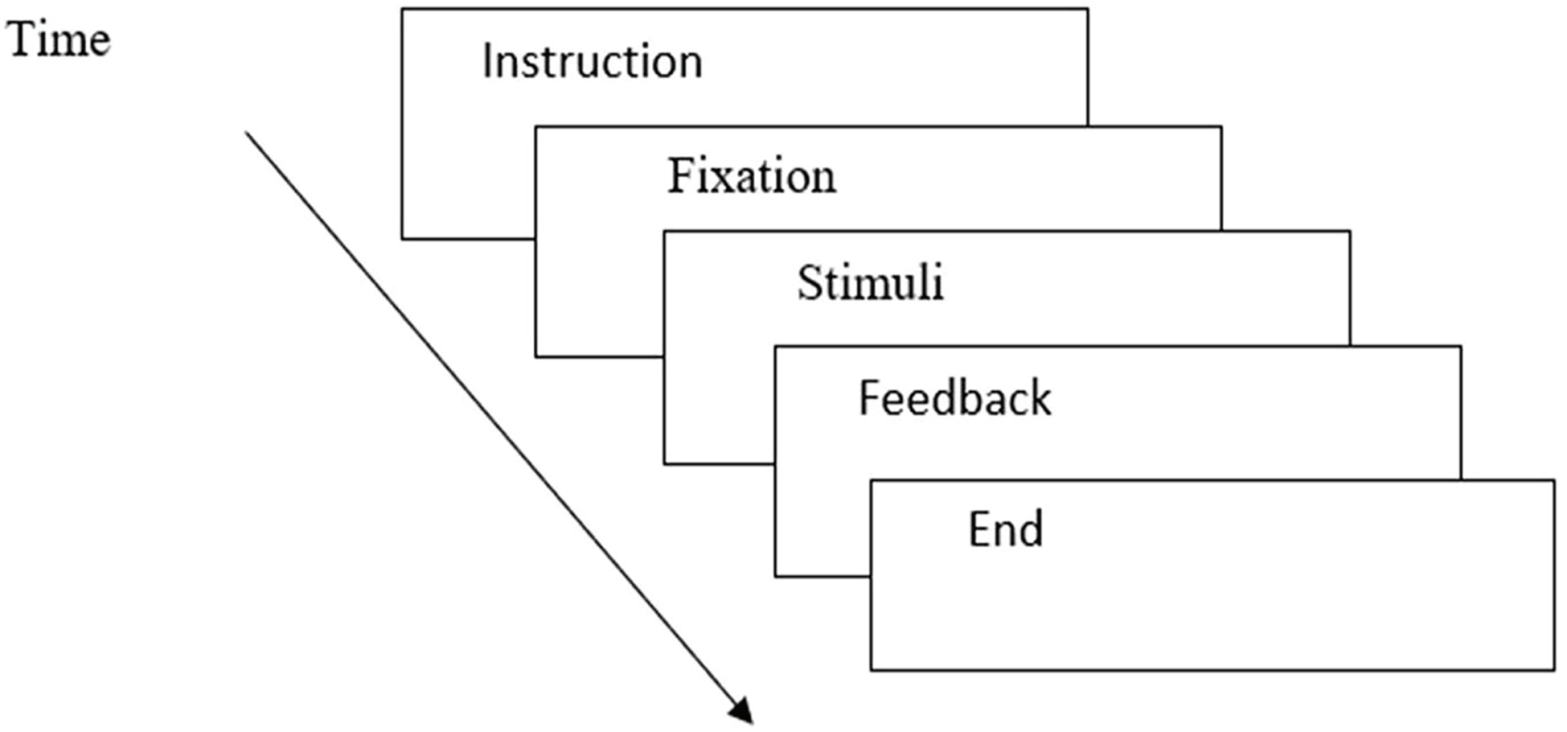
Procedure for the processing experiment by E-Prime.

All the participants were tested individually in a language laboratory. The participants read the sentences presented on a computer, using the software E-prime 2.0 (Schneider et al., 2012) in a word-by-word non-cumulative self-paced moving window paradigm (Just et al., 1982). The stimuli and the fillers were arranged in the same block (Figure 1).

At the beginning of the experimental session, the participants received five practice trials to familiarize themselves with the self-paced reading technique. Each sentence started with an asterisk to indicate the place that the first word would appear and a set of dashes, each representing a letter in the sentence. The sentence was presented word by word; the first screen looked like this: * --- -------- -- --------- ---- -- ----. The participant pressed a “space” bar to get the first word. After the participant finished reading it, (s)he pressed the “space” bar again, and then the first word was replaced by a set of dashes, and then the second word appeared to its right. An example was shown below. Delays in pushing the button indicated the processing difficulties of the previous “region of interest” or the fragment of the sentence.

* Bill ----- --- -------- ----- -- --- ---------.

* ---- *loved* --- -------- ----- -- --- ----------.

* ---- ----- *the* -------- ----- -- --- ----------.

*---- ----- --- *innocent* ----- -- --- ----------.

*---- ----- --- -------- *child* -- --- ----------.

*---- ----- --- -------- ----- *in* --- ----------.

*---- ----- --- -------- ----- -- *the* ----------.

*---- ----- --- ---- ---- ----- -- --- *playground*.

This process was repeated until the end of the sentence was finished. Then a yes/no comprehension question appeared, which checked the participant’s comprehension of the filler sentence. The participants answered the comprehension question as quickly and accurately as possible by pressing the “F” key for yes, and the “J” key for no. Feedback on accuracy was given for comprehension questions. E-prime automatically randomized the order of presentation of sentences for each participant and recorded all button presses to measure reading times with millisecond accuracy. The participants can pause to have a break if they needed one. Most of the participants finished the task in half an hour.

The self-paced reading technique has advantages in examining incremental language processing without the danger of potential confound from other retrieval or control processes present in many offline grammaticality judgment and production tasks (Jiang, 2013, p. 171). Furthermore, the possibility of using metalinguistic or explicit knowledge would also be minimized. This method allows one to measure the reading time for any word of a sentence (Jiang, 2013, p. 171).

### Data Analyses

Different from many sentence processing studies in the area of tense-aspect (e.g., Yap et al., 2009; Chan, 2012), the data for this self-paced reading experiment were analyzed *via* the Mixed Effects Models in R package instead of using ANOVA test in SPSS. All the participants scored 90% or above in the comprehension questions, so no participants were excluded in this study. However, the items that the participants wrongly comprehended and extreme reaction times (RTs) shorter than 100 ms or longer than 2,500 ms per word were discarded. These criteria led to the exclusion of 0.59, 1.36, and 1.26% of data points for the English native speakers, low-proficiency-level learners, and high-proficiency-level learners, respectively. All fillers were excluded from analysis.

Following Chan (2012) and Just and Carpenter (1980), separate analyses at four-word regions were conducted: the critical verb (V), the first word following the verb (V + 1) to capture spillover effects, the second word following the verb (V + 2) to assess further downstream effects among the L2 English learners, and, finally, the sentence final (SF) word to investigate sentence wrap-up effect.

The data were analyzed by performing Mixed Effects Model analysis of the relationship between groups, lexical aspect, and tense-aspect *via* R package *lme 4 1.1–14* (Bates et al., 2015) in R (R Core Team, 2019). As fixed effects, group (NS vs. CH_L vs. CH_H), lexical aspect (activity vs. state vs. achievement), and tense-aspect (PAST vs. Progressive) were entered into the model. As random effects, we had intercepts for subjects and items, as well as by-subject and by-item random slopes. The dependent variable is reaction time. The visual inspection of Q-Q plots and plots of residuals revealed no obvious deviations from homoscedasticity or normality after exclusion of the extreme data by model-based trimming. *P*-values were obtained by likelihood ratio tests of the full model with the effect in question against the model without the effect in question. When significant effects or main effects were found, *post hoc* of simple main effects was realized, using R package “lsmeans” (Lenth, 2014) to conduct further pairwise comparisons, using Tukey’s adjustment.

## Results

The results focus on the tests on the effects of the lexical aspect of verb predicates and learner’s L2 proficiency level in the past tense and the progressive processing. Table 5 shows a descriptive overview of the mean unadjusted reading times per word in milliseconds by lexical aspect in the past tense for three groups at the critical region (V).

**Table 5:** Mean and SD reaction times (ms) for past tense.

	State		Activity		Achievement	
	*M*	*SD*	*M*	*SD*	*M*	*SD*
NS	1,047	229	882	235	849	267
CH_H	1,183	278	1,080	326	877	196
CH_L	1,263	262	1,135	265	868	237

Visual inspection of the RT distribution revealed that the English native speakers exhibited the shortest reading times across the board than both the Mandarin EFL learners with a higher proficiency level and a lower proficiency level. The participants in all the groups read achievements faster than activity verbs and states in the past tense.

Table 6 shows a descriptive overview of the mean unadjusted reading times per word in millisecond by lexical aspect in progressive aspect for three groups at the critical region (V).

**Table 6:** Mean and SD reaction times (ms) for progressive aspect.

	State		Activity		Achievement	
	*M*	*SD*	*M*	*SD*	*M*	*SD*
NS	1,098	237	895	256	946	253
CH_H	1,180	317	907	274	1,011	346
CH_L	1,237	282	903	193	1,130	303

The English native speakers also exhibited the shortest reading times across the three lexical aspect classes than both the EFL learners with a higher proficiency level and a lower proficiency level. The participants in all the groups read activity verbs faster than achievement verbs and states in progressive aspect marking.

A Linear Mixed Effects Model analysis was performed to examine the main effects and interactions at four regions: critical region (V), post-critical region (V + 1), second word after the critical region (V + 2), and sentence final region (SF). Results are presented in the following by word region accordingly.

### Overall Results

First, to evaluate the effect of the lexical aspect of verb predicates in the tense-aspect processing by the three groups of the participants in both past tense and progressive marking conditions, the data in Tables 5 and 6 were submitted to a Linear Mixed Effects Model analysis with tense-aspect (past vs. progressive), lexical aspect (activity vs. achievement vs. state), and group (NS vs. CH_L vs. CH_H) as fixed effect factors. To keep the random-effects structure maximal (Barr et al., 2013), we included by-participants and by-items random slopes and their intercepts for all the relevant fixed effects.

The critical region of the verb is the main focus of the research (Models and results are in the Supplementary Material). A significant main effect of lexical aspect [*χ*^2^(2) = 128.21, *p* < 0.0001] was found. And the interaction between lexical aspect and tense-aspect was significant [*χ*^2^(2) = 77.92, *p* < 0.0001]. The interaction between lexical aspect, group, and tense-aspect was significant [*χ*^2^(4) = 67.53, *p* < 0.0001] as well. The Linear Mixed Effects Model Test at the post-critical region (a word after the verb) revealed a significant main effect of lexical aspect [*χ*^2^(2) = 551.8, *p* < 0.0001], and the interaction between lexical aspect, group, and tense-aspect was significant [*χ*^2^(4) = 68.1, *p* < 0.0001]. Similarly, at the region of second word after the critical region (V + 2) and sentence final region (SF), a significant main effect of lexical aspect [*χ*^2^(2) = 617.23, *p* < 0.0001] and [*χ*^2^(2) = 594.4, *p* < 0.0001] was found, respectively. This indicates the spillover effect of lexical aspect from the critical region.

### Past Tense

Next, this research evaluates the effect of the lexical aspect of verb predicates in the tense-aspect processing by the three groups of the participants at the critical region in the past tense. The data in Table 5 were submitted to a Linear Mixed Effects Model analysis with lexical aspect (activity vs. achievement vs. state) and group (NS vs. CH_L vs. CH_H) as fixed effect factors. To keep the random-effects structure maximal (Barr et al., 2013), we included by-participants and by-items random slopes and their intercepts for all the relevant fixed effects.

**Figure 2:**
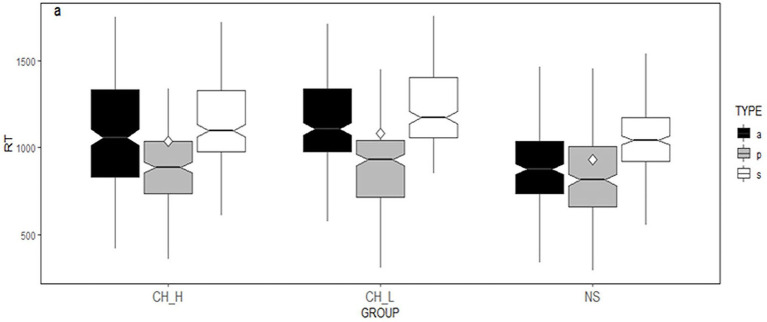
Box plots of the corresponding RTs by lexical aspect in the past tense at the critical region for the three groups. a, activity verbs; p, achievement verbs; and s, state verbs.

Figure 2 plots the corresponding RTs by condition and word region for each language group. The y-axis has been adjusted to the same scale for a direct comparison across groups. Each box describes the lower quartile, median, and upper quartile. The white dot represents the mean value of RTs by lexical aspect in the past tense.

The Linear Mixed Effects Model Test at the critical region revealed a significant main effect of lexical aspect [*χ*^2^(2) = 76.08, *p* < 0.001]. More importantly, the interaction between lexical aspect and group was significant as well (*χ*^2^(4) = 47.76, *p* < 0.001).

To explore the observed interaction between group and lexical aspect, a follow-up simple main effect of lexical aspect across the three groups was conducted in the critical word region. For the native speaker participants, there is a significant difference between achievements and states (*β* = 0.22924, SE = 0.0244, *t* = 9.407, *p* < 0.0001). Similarly, the lower-level Chinese participants read achievements significantly faster than states (*β* = 0.35951, SE = 0.0247, *t* = 14.545, *p* < 0.0001). The higher-proficient Chinese participants performed the same way (*β* = 0.27659, SE = 0.0246, *t* = 11.255, *p* < 0.0001). Namely, achievements are read significantly faster than states.

For achievement verbs, there is no significant difference observed between the groups (*ps* > 0.05). However, for states, significant differences are observed between CH_H and CH_L (*β* = 0.07291, SE = 0.0275, *t* = 2.653, *p* = 0.0094), and CH_H and NS (*β* = 0.10049, SE = 0.0291, *t* = 3.453, *p* = 0.0009), and CH_L and NS (*β* = 0.17340, SE = 0.0240, *t* = 7.216, *p* < 0.0001). As for activity verbs, significant differences are observed between CH_H and CH_L (*β* = 0.07, SE = 0.02, *t* = 2.69, *p* = 0.0084), and CH_H and NS (*β* = 0.18, SE = 0.0289, *t* = 6.371, *p* < 0.0001), and CH_L and NS (*β* = 0.25665, SE = 0.0237, *t* = 10.834, *p* < 0.0001).

For the sentences in past tense marking, the Linear Mixed Effects Model Test at the post-critical region revealed a significant main effect of lexical aspect [*χ*^2^(2) = 443.89, *p* < 0.001] and the interaction between lexical aspect, group, and tense-aspect was significant [*χ*^2^(2) = 151.89, *p* < 0.001]. Similarly, at the region of the second word after the critical region and sentence final region (SF), a significant main effect of lexical aspect [*χ*^2^(2) = 535.37, *p* < 0.001] and [*χ*^2^(2) = 508.44, *p* < 0.001] was found, respectively. This indicates the spillover effect of lexical aspect from the critical region.

### Progressive Aspect

**Figure 3:**
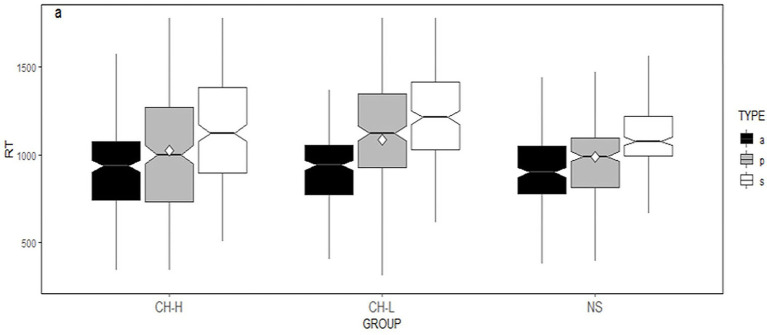
Box plots of the corresponding RTs by lexical aspect in the progressive aspect at the critical region for the three groups. a, activity verbs; p, achievement verbs; and s, state verbs.

Figure 3 plots the corresponding RTs by condition and word region for each language group in the progressive marking. For the sentences marked with progressive aspect, the Linear Mixed Effects Model Test at critical region revealed a significant main effect of lexical aspect [*χ*^2^(2) = 69.78, *p* < 0.0001]. Also, the main effect of the group was significant [*χ*^2^(2) = 49.16, *p* < 0.0001]. More importantly, the interaction between lexical aspect and group was significant [*χ*^2^(4) = 27.39, *p* < 0.0001].

A follow-up simple main effect of lexical aspect across the three groups was conducted in the critical word region to explore the observed interaction between group and lexical aspect. Significant differences between the activity verbs and states are found in all the three groups (NS participants: *β* = 0.21184, SE = 0.0252, *t* = 8.398, *p* < 0.0001; CH_H: *β* = 0.23775, SE = 0.0255, *t* = 9.333, *p* < 0.0001; CH_L: *β* = 0.30067, SE = 0.0254, *t* = 9.328, *p* < 0.0001).

No significant difference is observed in activity verbs marked with progressive aspect between the three groups (*ps* > 0.05). However, for states, significant differences are observed between CH_H and CH_L (*β* = 0.0999, SE = 0.0244, *t* = 0.2325, *p* = 0.0015). As for achievement verbs, significant differences are observed between CH_H and CH_L (*β* = 0.14401, SE = 0.0242, *t* = 5.941, *p* < 0.0001; and CH_L and NS *β* = 0.17554, *SE* = 0.0242, *t* = 7.249, *p* < 0.0001).

For the sentences in the progressive marking, the Linear Mixed Effects Model Test at the post-critical region revealed a significant main effect of lexical aspect [*χ*^2^(2) = 303.83, *p* < 0.0001] and the interaction between lexical aspect, group, and tense-aspect was significant [*χ*^2^(4) = 27.42, *p* < 0.0001]. Similarly, a significant main effect of lexical aspect was found at the region of the second word after the critical region [*χ*^2^(2) = 304.42, *p* < 0.0001] and at the sentence final region [*χ*^2^(2) = 302.27, *p* < 0.0001]. This indicates the spillover effect of lexical aspect from the critical region.

In sum, the results show that the lexical aspect of verb predicates plays an important role in the processing of both English past tense and progressive aspect marking by all three groups. The prototypical associations of English tense-aspect categories predicted in the Aspect Hypothesis, such as achievement verbs with past tense and activity verbs with progressive, can engender shorter reading times than non-prototypical associations for both the native speakers and the L2 learners.

Although Chinese learners’ native language does not encode tense grammatically, and lexical aspect also interacts with grammatical aspect differently from that in English, results show that it does not affect their processing of prototypical exemplars (the achievement verbs in past tense marking). That is, there is no significant difference between the native speakers and the L1 Chinese learners in their processing of achievement verbs with past tense marking. However, significant differences exist in the processing of non-prototypical items. Although Mandarin Chinese also encodes the progressive aspect grammatically just like English, no significant difference exists between the native speaker participants and the L1 Chinese participants when they are processing activity verbs with progressive marking. However, when they are processing non-prototypical association (states with progressive marking), a significant difference was observed between the native speakers and the L1 Chinese learners.

The results indicate that there is no effect of language proficiency on learners’ processing of prototypes in L2 tense-aspect marking. For both L2 proficiency levels, there is no significant difference between the reading time for the prototypical association of activity verbs with progressive marking and associations of achievement verbs and state verbs with past tense. However, there is a significant difference between the lower-proficiency learners and the higher-proficiency learners in their processing of non-prototypes (states and activities with past tense, and achievements and states with progressive making) in the L2 tense-aspect marking.

## Discussion

### The Effect of Lexical Aspect on Tense-Aspect Processing

The first purpose of this experiment is to test whether the lexical aspect of verb predicates to which tense-aspect marking is attached has an effect on L2 English tense-aspect processing and whether the prototypes stipulated in the Aspect Hypothesis can facilitate processing. Results show that there is a significant main effect of lexical aspect in the processing of both English past tense and progressive aspect marking in all the participant groups. The interaction between lexical aspect and tense-aspect marking is also significant. Specifically, the prototypical combinations of English tense-aspect marking predicted in the Aspect Hypothesis, i.e., achievement verbs in past tense and activity verbs with progressive marking, can engender shorter reading time than non-prototypical combinations (i.e., state and activity verbs with the past tense, achievement and state verbs with progressive making) for native speakers. The Chinese learners of English in this study show a similar processing pattern. Their reading time for past tense and progressive aspect is also related to the lexical aspect of verbs.

This result is in line with the findings from Yap et al. (2009) and Madden and Zwaan (2003). The English native speakers in our study and Cantonese native speakers in Yap et al. (2009) are observed to have significantly faster processing speed in accomplishment with past tense and activity verbs with progressive than other category combinations. There are interactions between grammatical aspect and lexical aspect in tense-aspect processing. Therefore, the results indicated that verb type contributes to aspectual asymmetry during language processing. In other words, verb types play different roles in the processing of events. This result is also in line with Madden and Zwaan (2003), which found the perfective facilitation effect for accomplishment verbs in past tense marking, because accomplishments, just like achievements, are telic and compatible with perfective marking. The findings from our study and previous studies have indicated that lexical aspect and grammatical aspect contribute to the reader’s mental model of a situation. This current online processing result also supports the results from most production studies. For example, in a longitudinal study of two Korean learners of English, Lee (2001) found out that the past tense was predominantly associated with telic predicates before emerging in other atelic contexts. Therefore, consistent with the prediction of the Aspect Hypothesis, the prototype effect is often observed in tense-aspect processing and acquisition.

The current study found that prototypical associations, such as Achievement PAST and Activity PROG, yield shorter RTs than less prototypical associations among native speakers. However, Chan (2012) found that such online processing biases did not reach statistical significance for native speakers. In Chan (2012, p. 94), the Chinese participants processed State PAST significantly faster than Activity PAST, which goes against the prediction of the prototype hypothesis. No further significant RT differences between State PAST and Achievement PAST were found. In contrast, in the current study, our Chinese participants processed prototypical association of Achievement PAST significantly faster than State PAST. The discrepancy with regard to the processing of past tense might have been caused by the research design. Just as previous research, such as Pinker and Prince (1994) and Housen (2002) argued, the processing mechanisms of regular past and irregular past may be different (see Shirai, 2019, p. 60–64 for a review of L2 literature on the regular-irregular debate). In fact, Housen (2002) claimed that the effect of lexical aspect is stronger for regular morphology than irregular morphology because learners mainly rely on an associative or rote-learning mechanism in the acquisition and use of the irregular morphology, while, for the regular morphology, they tend to rely on productive, symbol-manipulating rule application. The current study does not follow Chan (2012), which includes both regular and irregular past verbs. Instead, all verbs in past tense in our study are regular ones. This might be a reason that the results in the current study exhibited processing asymmetry among native speakers but not in Chan (2012). If Housen’s claim is correct, it makes sense that the present study, which only looked at regular past but not irregular past tense, showed a stronger effect of lexical aspect than Chan (2012), which included both regular and irregular past. It should be noted, however, that other L2 studies (Rocca, 2002; Chan et al., 2012) did not support Housen’s claim, and both regular and irregular morphology was influenced by lexical aspect (Shirai, 2019). The interaction of morphological regularity and lexical aspect needs to be further studied both in acquisition and processing.

In terms of processing the non-prototypical association of progressive with states, both proficiency levels of Chinese L2 learners in the current study were found to process stative progressives much more slowly than activity progressives, while, in Chan (2012), stative progressives were processed faster than activity progressives, although the difference was not statistically significant. It is not clear why this discrepancy is observed. One possibility is that the two studies did not use the same list of verbs – the overlap was about 80% since we had to include different verbs to match neighborhood density, etc. Another discrepancy worth mentioning is that no significant difference was observed in RTs in processing stative progressives between the English native speakers and the Chinese L2 learners in Chan (2012), and between the English native speakers and the higher proficiency group in the current study, while a significant difference was found between the native speakers and the lower proficiency group in our study. In other words, native-like performance was possible for higher-level learners in the current study but not by lower-level learners. This result again suggests that language learning is a developmental phenomenon. The more complex and weaker association between a form and its meaning, the longer time is needed for learners. This observation is in line with previous research: Fuchs and Werner (2018) found that beginning/intermediate level learners rarely use progressive with states while advanced learners in Dose-Heidelmayer and Götz (2016) did. With more target language input and experience with the language, higher-proficiency L2 learners, we suggest, have become more flexible with less prototypical combinations of lexical aspect and the progressive aspect and can process the progressive aspect just like the native speakers do.

Then how can the results that the prototypical associations of tense-aspect categories engender shorter reading time be explained? Yap et al. (2009) proposed semantic compatibility, which is believed to be a basic cognitive principle, to account for their findings. The Cantonese perfective marker -*zo* with telic accomplishment verbs is just like the English (perfective) past marker -*ed* with telic achievement verbs. The semantic value of the aspectual morpheme -*ed* matches with the semantic value of telic verbs. They are bounded, punctual. The Cantonese imperfective marker -*gan* with atelic activity verbs is just like the English progressive marker -*ing* with atelic activity verbs. There is semantic compatibility between the English progressive marker -*ing* with atelic activity verbs because they are not bounded, punctual, but dynamic.

The result can also be explained from the usage-based account of language acquisition, which holds that various psychological factors underlying the online processing of constructions. Factors, such as frequency, type-token frequency distribution, contingency, and semantic prototypicality are crucial to L2 processing (Ellis et al., 2016a, p. 43). Both the native and non-native speakers are shown to be sensitive to statistical patterns of use. Generally speaking, the most frequent verb types are closely associated with the special construction, and they have a strong contingency. It is the contingency of the verb and the special tense-aspect construction, which gives special, specific, readily accessible meanings; therefore, they are much easier to process. The processing involves semantics; that is, verbs that are more prototypical of the construction semantic meaning can cause greater activation. The frequency in production studies (e.g., Robison, 1995b) show that achievement verbs have a high frequency in the past tense construction and so do activity verbs in the progressive construction in native discourse (the Distributional Bias Hypothesis, see Andersen and Shirai (1996) for a crosslinguistic review of frequent combinations in native discourse). The argument is that the high frequency of certain verbs with special construction may generate prototypical meaning. Many other studies show that reading time is affected by collocational and sequential probabilities. Bod (2001), for example, employing a lexical decision task, found out that high-frequency three-word sentences such as “*I like it*” have shorter reading times than low-frequency sentences such as “*I keep it*” by native speakers.

These two accounts – the semantic account and the frequency account – are both possible. In fact, the frequency account could argue that semantics is irrelevant, and everything can be accounted for by the frequency of combination. This question has not been fully addressed in the literature on the AH, but the best attempt is made by Johnson and Fey (2006). In their elicited imitation study with L1 children, they contrasted the same verb in telic and atelic conditions (e.g., *roll a ball into a box* vs. *roll a ball on a box*) and found that prototypical combination is easier to produce, suggesting that the effect of telicity is not just frequency effect.

It is likely that the higher the frequency of the verb type is in the tense-aspect construction and the higher the contingency, the more accessible the tense-aspect construction is, and thus the faster the processing is made. However, more controlled studies are in order to tease them apart.

### The Effect of the Learners’ L2 Proficiency Level on Tense-Aspect Processing

The second purpose of this experiment is to explore whether learners’ L2 proficiency level influences L2 tense-aspect processing. Results suggest that the interaction between lexical aspect, tense-aspect marking, and group is significant. There is a significant main effect of group in both past tense and progressive aspect marking in all the regions examined. The prototypical associations of English tense-aspect categories, such as achievement verbs with past tense and activity verbs with progressive, can engender shorter reading times than non-prototypical associations for both the native speakers and the Chinese learners of English in this study. For all the native English speakers and the L2 learners at both proficiency levels, their reading time for past tense and progressive aspect is also related to the lexical aspect of verbs. The reading time for the prototypical associations of achievement verbs and past tense marking and the associations of activity verbs with progressive marking are much shorter than that in the less prototypical associations of activity verbs and state verbs with past tense and associations of achievement verbs and state verbs with past tense. Therefore, there is no effect of the L2 proficiency level on learners’ processing of prototypes in L2 tense-aspect marking.

However, the results reveal that L2 proficiency level effect is observed in its processing of non-prototypes in the L2 tense-aspect marking. Significant differences were observed between learners with a lower-L2-proficiency level and a higher-L2-proficiency level, and, also, between the lower-L2-proficiency learners and native speakers in the processing of state verbs in past tense marking (see Figure 2). For the processing of state verbs in the progressive marking, though no significant difference is observed between the learners at two L2 proficiency levels, there is a significant difference between the lower-L2-proficiency level of the learners and the native speakers (see Figure 3).

The effect of proficiency observed in the current study can be explained from the usage-based account of language acquisition, which holds that practice promotes proficiency (Ellis et al., 2016b). The more frequently learners experience something, the stronger their memory for it is, the easier it is accessed, and, therefore, the shorter time for processing. As noted in the previous section, according to the findings in the production research, the prototypes are frequent constructions in the input (Andersen and Shirai, 1996). The L2 learners acquire them from the very beginning; therefore, with enough input and practice, the prototypes are entrenched in their L2 knowledge. The more they become associated in learners’ minds with more time experiencing conjunctions of features, the more they subsequently affect processing and categorization (Ellis et al., 2016a). However, for the non-prototypical types, the Chinese L2 learners of English with a lower-proficiency level are found to suffer from greater problems in tense-aspect processing. They could not process states with the past tense and the progressive marking as fast as the learners with a higher-proficiency level and the English native speakers. This suggests the effect of usage-based learning supported by processing frequency, which eventually helps advanced learners process tense-aspect like native speakers. Even though the L1 Chinese speakers are shown to be less sensitive to morphological processing in L2 (Ellis and Sagarra, 2010) because of impoverished morphology in their L1, still native-like performance seems possible for the advanced learners. This interplay of L1 effect and processing frequency must be addressed in the future research where two L1 groups with different morphological profiles (e.g., Chinese vs. Spanish) are tested.

The usage-based psycholinguistic research states that our language processing is sensitive to the statistical regularities of language experience at every level of structure (Ellis et al., 2016a, p. 279). In language processing, we argue that prototypes are less likely to be influenced by knowledge about learners’ L1, because these prototypical constructions are Zipfian in their verb type – construction constituency in usage. Psychological theories related to the statistical learning of categories also make clear that these are important factors that promote learning. In contrast, the non-prototypical verb-construction categories have low entrenchment and contingency, so language users are more likely to be influenced by knowledge of these usage statistics in their L1. Ellis et al. (2016a) pointed out that learners whose L1 is similar to English exhibited more target-like verb argument construction (VAC) associations than those whose L1 is not. Taken together, the effect of learners’ L1 on tense-aspect processing requires future research into online studies of bilingual tense-aspect processing.

Finally, a note on native speakers’ processing of tense-aspect markers is in order. The present study clearly shows the importance of lexical aspect in tense-aspect processing. As briefly mentioned earlier, Madden and Zwaan (2003) found the facilitation effect of perfective (i.e., past tense) marking in English and argued it was due to compact representation of perfective aspect in comparison to more diffuse representation of imperfective aspect (be V-ing). However, they only used accomplishment verbs, which are telic and compatible with perfective aspect, in their experiment. Yap et al. (2009) found that it is not perfective advantage itself but the interaction of lexical and grammatical aspect, showing progressive imperfective is processed faster with atelic activities than with telic verbs in Cantonese. The present study replicates this interaction in English native speaker’s processing, thus suggesting that interaction of lexical and grammatical aspect is the key to faster processing of perfective with accomplishment verbs in Madden and Zawaan’s experiment. The interaction of lexical and grammatical aspect/tense is ubiquitous (Shirai, 2011).

## Conclusion

This study investigates whether the lexical aspect of verbs and the learners’ L2 proficiency level have an impact on their tense-aspect processing. A psycholinguistic online processing experiment (self-paced reading) was conducted among the L2 Chinese learners of English at two proficiency levels and native English speakers. The results show the following: (1) the lexical aspect of verbs influences L2 learners’ and native speakers’ sentence processing of prototypes, namely achievement verbs in past tense marking and activity verbs in progressive marking can engender shorter processing time than non-prototypical combinations by both L2 learners and English native speakers; (2) No effect of L2 proficiency level is observed on the learners’ processing of prototypes of L2 tense-aspect marking. However, the effects of the proficiency level are observed in their processing of non-prototypes in the L2 tense-aspect marking. The online processing results support the prediction of the Aspect Hypothesis.

## Data Availability Statement

The raw data supporting the conclusions of this article will be made available by the authors, without undue reservation.

## Ethics Statement

The studies involving human participants were reviewed and approved by School of Foreign Languages, Research Ethical Review Board, Hunan University. The patients/participants provided their written informed consent to participate in this study.

## Author Contributions

XZ and YS conceived and designed the experiment, while XZ was a visiting scholar at Case Western Reserve University (2016–2017). XZ implemented the experiment, collected, and analyzed the data and wrote the manuscript. XC and YS contributed to the revision of the article. All authors contributed to the article and approved the submitted version.

### Conflict of Interest

The authors declare that the research was conducted in the absence of any commercial or financial relationships that could be construed as a potential conflict of interest.
