# Supplementary material for: Lexical and Grammatical Aspect in On-line Processing of English Past Tense and Progressive Aspect by Mandarin Speakers
Source: Front Psychol. 2021 Jun 10;12:661923. doi: 10.3389/fpsyg.2021.661923 (PMC8222903; doi:10.3389/fpsyg.2021.661923)
Supplement: Supplementary file 1 [file Table_1.pdf]

Supplementary Table 1: Length, frequency, and orthographic neighborhood density of verbs in past tense and progressive

	Past tense				Progressive		
	Len.	Freq.	Orth. N.		Len.	Freq.	Orth. N.
agreed	6	25653	1	beginning	9	10825	0
arrived	7	26891	1	believing	9	30523	1
attended	8	11707	0	breaking	8	6709	1
believed	8	28434	3	checking	7	20017	0
called	6	229335	6	choosing	8	10171	0
considered	10	15583	0	expecting	9	9266	0
disliked	8	1169	1	feeling	7	34491	4
dropped	7	21542	5	finding	7	34669	3
encouraged	10	6041	1	forgetting	10	3385	0
enjoyed	7	10815	0	getting	7	160942	7
escaped	7	3770	2	going	5	677128	1
finished	8	16376	2	having	6	163153	7
fixed	5	1508	4	helping	7	31018	1
hated	5	9848	4	hoping	6	24442	2
helped	6	35387	3	living	6	54090	6
killed	6	22295	5	loving	6	4212	4
learned	7	33315	1	ordering	8	3638	0
liked	5	28247	4	playing	7	70342	3
locked	6	4510	11	preferring	10	1576	0
loved	5	32572	6	reaching	8	17578	4
needed	6	55130	2	reading	7	44510	5
owned	5	8041	1	running	7	70667	4
preferred	9	4870	0	saying	6	143800	6
pushed	6	19207	4	sending	7	18667	8
questioned	10	4428	1	shouting	8	6606	3
smashed	7	1474	3	showing	7	31913	4
touched	7	12129	2	training	8	5020	3
trained	7	1751	4	wishing	7	3988	2
wanted	6	156136	4	worrying	8	2023	0
washed	6	3775	8	writing	7	37622	2

Note: Len, Length; Freq., frequency; Orth. N., orthographic neighborhood density
