# Supplementary material for: Lexical and Grammatical Aspect in On-line Processing of English Past Tense and Progressive Aspect by Mandarin Speakers
Source: Front Psychol. 2021 Jun 10;12:661923. doi: 10.3389/fpsyg.2021.661923 (PMC8222903; doi:10.3389/fpsyg.2021.661923)
Supplement: Supplementary file 2 [file Data_Sheet_1.PDF]

Stimuli for Online Processing of English Past Tense and Progressive Aspect

STA: state

ACT: activity

ACH: achievement

PAST

1.	Bill loved the innocent child in the playground.	STA
2.	Bill helped the innocent child in the playground.	ACT
3.	Bill killed the innocent child in the playground.	ACH
4.	Henry hated the naughty boy from a local school.	STA
5.	Henry trained the naughty boy from a local school.	ACT
6.	Henry called the naughty boy from a local school.	ACH
7.	Ann liked the bike while going up the hill.	STA
8.	Ann pushed the bike while going up the hill.	ACT
9.	Ann smashed the bike while going up the hill.	ACH
10.	Joe liked the hot coffee and burned his tongue.	STA
11.	Joe enjoyed the hot coffee and burned his tongue.	ACT
12.	Joe touched the hot coffee and burned his tongue.	ACH
13.	Steve owned a language class before he travelled abroad.	STA
14.	Steve attended a language class before he travelled abroad.	ACT
15.	Steve finished a language class before he travelled abroad.	ACH
16.	Karen wanted five kittens in her house.	STA
17.	Karen washed five kittens in her house.	ACT
18.	Karen locked five kittens in her house.	ACH
19.	Sam wanted the big television in the mall.	STA
20.	Sam played the big television in the mall.	ACT
21.	Sam smashed the big television in the mall.	ACH
22.	Sue believed Lynn about her studies.	STA
23.	Sue encouraged Lynn about her studies.	ACT
24.	Sue questioned Lynn about her studies.	ACH
25.	John agreed at the airport to fly planes.	STA
26.	John trained at the airport to fly planes.	ACT
27.	John arrived at the airport to fly planes.	ACH
28.	Chris preferred the expensive pair of shoes at the store.	STA
29.	Chris considered the expensive pair of shoes at the store.	ACT
30.	Chris dropped the expensive pair of shoes at the store.	ACH
31.	Susan loved the red guitar at the musical fair.	STA

32. Susan played the red guitar at the musical fair.	ACT
33. Susan smashed the red guitar at the musical fair.	ACH
34. Henry liked the children in front of the school building.	STA
35. Henry trained the children in front of the school building.	ACT
36. Henry questioned the children in front of the school building.	ACH
37. She preferred some huge carrots from her vegetable garden.	STA
38. She washed some huge carrots from her vegetable garden.	ACT
39. She finished some huge carrots from her vegetable garden.	ACH
40. They disliked the woman at the karaoke bar around the corner.	STA
41. They pushed the woman at the karaoke bar around the corner.	ACT
42. They touched the woman at the karaoke bar around the corner.	ACH
43. They preferred their honeymoon in New Zealand.	STA
44. They enjoyed their honeymoon in New Zealand.	ACT
45. They finished their honeymoon in New Zealand.	ACH
46. He believed his teacher from karate class.	STA
47. He helped his teacher from karate class.	ACT
48. He called his teacher from karate class.	ACH
49. David hated the brown dog in his neighborhood.	STA
50. David trained the brown dog in his neighborhood.	ACT
51. David killed the brown dog in his neighborhood.	ACH
52. Joan loved the chocolate cookies last Christmas.	STA
53. Joan enjoyed the chocolate cookies last Christmas.	ACT
54. Joan touched the chocolate cookies last Christmas.	ACH
55. Mary believed some knowledge from her math teacher.	STA
56. Mary learned some knowledge from her math teacher.	ACT
57. Mary questioned some knowledge from her math teacher.	ACH
58. Bob wanted three gold medals at the Olympic Games.	STA
59. Bob enjoyed three gold medals at the Olympic Games.	ACT
60. Bob dropped three gold medals at the Olympic Games.	ACH
61. Peter owned the white tent used for the wedding.	STA
62. Peter pushed the white tent used for the wedding.	ACT
63. Peter touched the white tent used for the wedding.	ACH
64. Tom needed travel books written by a famous author.	STA
65. Tom enjoyed travel books written by a famous author.	ACT
66. Tom finished travel books written by a famous author.	ACH
67. He hated the yellow car in the parking lot.	STA
68. He fixed the yellow car in the parking lot.	ACT
69. He locked the yellow car in the parking lot.	ACH
70. Mary preferred colorful hats on her birthday party.	STA
71. Mary considered colorful hats on her birthday party.	ACT
72. Mary smashed colorful hats on her birthday party.	ACH

PROG

1.	Tom is hoping to win the game on Saturday.	STA
2.	Tom is training to win the game on Saturday.	ACT
3.	Tom is beginning to win the game on Saturday.	ACH
4.	Ben is believing what the book said about robots.	STA
5.	Ben is checking what the book said about robots.	ACT
6.	Ben is forgetting what the book said about robots.	ACH
7.	Mary is expecting Tony to study abroad during the summer.	STA
8.	Mary is helping Tony to study abroad during the summer.	ACT
9.	Mary is sending Tony to study abroad during the summer.	ACH
10.	Joan is worrying whether there are too many cars on the highway.	STA
11.	Joan is checking whether there are too many cars on the highway.	ACT
12.	Joan is finding whether there are too many cars on the highway.	ACH
13.	Sam is living with his cousin from Chicago.	STA
14.	Sam is playing with his cousin from Chicago.	ACT
15.	Sam is going with his cousin from Chicago.	ACH
16.	She is feeling that she could make a useful contribution.	STA
17.	She is shouting that she could make a useful contribution.	ACT
18.	She is saying that she could make a useful contribution.	ACH
19.	Lily is wishing for first place in the marathon.	STA
20.	Lily is running for first place in the marathon.	ACT
21.	Lily is reaching for first place in the marathon.	ACH
22.	Bob is preferring the original version of Cinderella.	STA
23.	Bob is reading the original version of Cinderella.	ACT
24.	Bob is getting the original version of Cinderella.	ACH
25.	Harry is loving his sister Mary's birthday present.	STA
26.	Harry is playing his sister Mary's birthday present.	ACT
27.	Harry is breaking his sister Mary's birthday present.	ACH
28.	Joy is loving the material about aliens landing on Earth.	STA
29.	Joy is writing the material about aliens landing on Earth.	ACT
30.	Joy is ordering the material about aliens landing on Earth.	ACH
31.	Jessica is living up the hill near the church.	STA
32.	Jessica is running up the hill near the church.	ACT
33.	Jessica is going up the hill near the church.	ACH
34.	Tom is living the vibrant city life of New York City.	STA
35.	Tom is writing the vibrant city life of New York City.	ACT
36.	Tom is finding the vibrant city life of New York City.	ACH
37.	Peter is expecting a French novel at Christmas.	STA
38.	Peter is reading a French novel at Christmas.	ACT
39.	Peter is beginning a French novel at Christmas.	ACH
40.	Susan is loving the fruit salad at the fast food restaurant.	STA

41.	Susan is choosing the fruit salad at the fast food restaurant.	ACT
42.	Susan is ordering the fruit salad at the fast food restaurant.	ACH
43.	Amy is expecting her travel agent to find a cheaper ticket.	STA
44.	Amy is helping her travel agent to find a cheaper ticket.	ACT
45.	Amy is getting her travel agent to find a cheaper ticket.	ACH
46.	Daniel is loving the details published in this book.	STA
47.	Daniel is reading the details published in this book.	ACT
48.	Daniel is forgetting the details published in this book.	ACH
49.	Eric is loving the German book by a famous writer.	STA
50.	Eric is reading the German book by a famous writer.	ACT
51.	Eric is breaking the German book by a famous writer.	ACH
52.	Sarah is expecting her dog to get in the car.	STA
53.	Sarah is helping her dog to get in the car.	ACT
54.	Sarah is getting her dog to get in the car.	ACH
55.	Jack is loving the abstract painting for the art exhibition.	STA
56.	Jack is choosing the abstract painting for the art exhibition.	ACT
57.	Jack is ordering the abstract painting for the art exhibition.	ACH
58.	George is worrying the business plan for a new salon.	STA
59.	George is writing the business plan for a new salon.	ACT
60.	George is finding the business plan for a new salon.	ACH
61.	He is expecting Sally to move her belongings from the kitchen.	STA
62.	He is helping Sally to move her belongings from the kitchen.	ACT
63.	He is getting Sally to move her belongings from the kitchen.	ACH
64.	David is having a question about the universe.	STA
65.	David is writing a question about the universe.	ACT
66.	David is getting a question about the universe.	ACH
67.	Peter is loving the ambassador of Italy.	STA
68.	Peter is helping the ambassador of Italy.	ACT
69.	Peter is forgetting the ambassador of Italy.	ACH
70.	Mike is expecting dog food for his new puppy.	STA
71.	Mike is showing dog food for his new puppy.	ACT
72.	Mike is ordering dog food for his new puppy.	ACH

Filler Items

Filler 1

John did not get the Oscar because he is not a very good actor and there were better deserving candidates.

Did John win the Oscar? N

Filler 2

I got a lower grade on my paper because I made a careless error and the teacher expected better.

Were careless errors made on the paper? Y

Filler 3

Actors need to follow the instructions of the director during filming.

Do actors need to follow directions? Y

Filler 4

Every morning for breakfast Mary put milk in her bowl of cereal even though it would no longer be crunchy.

Does Mary put milk in her bowl of cereal? Y

Filler 5

He had to rent a tuxedo because the party was formal and very high class.

Was the party formal? Y

Filler 6

The restaurant was so dirty that the health inspector came and forced it to close.

Was the restaurant dirty? Y

Filler 7

His fever was high so we drove him to the hospital in the strange town.

Was the town strange? Y

Filler 8

At the airport gate, his belt buckle set off the metal detector and embarrassed us all.

Did the belt buckle set off the metal detector? Y

Filler 9

The phone company charges for long distance calls but not for local calls, which are free.

Does the phone company charge for long distance calls? Y

Filler 10

Before playing, the musical composer first wiped the keys of the piano at the beginning of the concert.

Did the musical composer wipe the piano keys? Y

Filler 11

Compared with others, the luxury car was far superior in terms of performance.

Does the luxury car perform poorly? N

Filler 12

Watching the tornado near my home I felt great terror but tried to keep calm.

Was there a tornado? Y

Filler 13

My new salary is more than double, if not triple what I used to make.

Is the new salary lower than the original? N

Filler 14

Although annoyed, I tried to keep my calm as she complained.

Was I happy that she complained? N

Filler 15

For years she was a singer in a band that used to play in this bar.

Was she a drummer in the band? N

Filler 16

The local farmer rode on his tractor where ever he went.

Does the farmer only ride his tractor on the farm? N

Filler 17

After writing a few sonnets he decided to become a poet and we enjoyed his work very much.

Did he write a few essays? N

Filler 18

The child was ashamed because her answer was not correct and she did not raise her hand again.

Did the child raise her hand again? N

Filler 19

The child yelled into the well and she could hear her voice echo a few times.

Did the child fall in the well? N

Filler 20

The frog caught a fly and another insect on its tongue.

Did the frog catch a bird? N

Filler 21

She enjoyed the college course because the young professor was interesting and helpful.

Was the professor boring? N

Filler 22

He lit candles and bought flowers to make the mood more romantic for their anniversary.

Did he buy a car? N

Filler 23

While in New York the unfaithful husband had an affair with another woman.

Did the husband have an affair? Y

Filler 24

As we got to know each other we felt a close bond growing between us.

Did they get to know each other? Y

Filler 25

It was getting very cold so I put on my coat and ski gloves.

Was it very cold? Y

Filler 26

I fell off the horse and knew that I should get back up on the saddle and try jumping again.

Did she fall off the horse? Y

Filler 27

My aunt just had a baby so now I have a new cousin to play with.

Was a baby born? Y

Filler 28

The students learned to follow the instructions of their teacher before being allowed to play with the blocks.

Did the student learn to follow instructions? Y

Filler 29

To throw the ball in the hoop you need to bend your elbow at the correct angle.

Do you need to bend you elbow to throw a ball in the hoop? Y

Filler 30

At first the movie was interesting but it had a strange ending which we could not comprehend.

Was the movie interesting at first? Y

Filler 31

Once pregnant we realized the cat was actually a female and not a male.

Was the cat pregnant? Y

Filler 32

The guests cried when the priest said, "I now pronounce you husband and wife.

Did the guests cry? Y

Filler 33

The body aches and high fever indicated that she had an illness that would not soon go away.

Did she have a fever? Y

Filler 34

His future bride was so angry that she broke off the engagement for an entire year.

Was the bride happy? N

Filler 35

The steamboat floated slowly down the river as the sun set in the distance.

Did the steam boat sink? N

Filler 36

They found the gun and arrested him for attempted murder and armed robbery.

Did they find a knife? N

Filler 37

The student looked for some paper and a sharp pencil to write with. Did the student look for an ink pen? N

Filler 38

Most of the year it is warm but it gets really hot in the summer months before school starts.

Is it cold most of the year? N

Filler 39

At the art gallery I admired the painting that had come from France.

Did the painting come from Germany? N

Filler 40

She was sick for a long time and we hoped for a quick recovery so she could go to the wedding.

Was she feeling well? N

Filler 41

The soup was so hot I burned my tongue and almost spit it back out.

Was the soup cold? N

Filler 42

Just the right spices made the sauce even more tasty and we all asked for seconds.

Did the sauce taste bad? N

Filler 43

The warring nations signed the peace treaty after many months of discussion.

Did the warring nations sign the treaty? Y

Filler 44

She assured me that he was a wonderful actor and that the movie was great.

Did she think the movie was great? Y

Filler 45

As soon as I had turned around my nephew threw the cereal on the floor and refused to eat.

Did the nephew throw the food on the floor? Y

Filler 46

They disagreed a lot and did not like the director after a few months.

Did they disagree a lot? Y

Filler 47

Carlos was the one who made the error in calculation.

Did Carlos make an error in calculation? Y

Filler 48

She informed us that it was a formal party and that we would need to dress up.

Did they need to dress up? Y

Filler 49

He was relieved that the hospital was only a few blocks away.

Was the hospital only a few blocks away? Y

Filler 50

The family hoped that the inspector would come and test the water.

Did the family want the inspector to test the water? Y

Filler 51

We read about a new local restaurant which served great food.

Does the restaurant serve great food? Y

Filler 52

On our way back home we noticed the metal roof had come off of the shed.

Did the shed have a wooden roof? N

Filler 53

When we entered the dining hall we saw the piano in the corner of the room.

Was there a guitar in the corner of the room? N

Filler 54

My uncle was pleased with the tractor he had bought at the state fair.

Did the uncle buy a car at the fair? N

Filler 55

The town people remembered the terror they had felt during the hurricane.

Were the town people happy about the hurricane? N

Filler 56

Last time, I had to take triple the amount of pain reliever in order to sleep.

Did the person drink wine to fall asleep? N

Filler 57

They kept searching for a superior carpenter to get the job done.

Were they searching for a plumber? N

Filler 58

She finally got a chance to see a good band play live music at the bar.

Did a terrible band play at the bar? N

Filler 59

It was impressive that she was calm through all those problems.

Was she anxious about her problems? N

Filler 60

I checked to make sure the correct ingredients were added.
Were incorrect ingredients added? N
Filler 61
We were interested in the talk but there was an echo in the room and we could not hear well.
Was it easy to hear the talk? N
Filler 62
The teacher explained to us how the insect mated and laid eggs.
Did the teacher explain how chickens lay eggs? N
Filler 63
An old friend of mine from college was a poet and was enjoying her work very much.
Did the friend dislike her work? N
Filler 64
They looked forward to the arrival of the professor to the university.
Did they look forward to the professor's arrival? Y
Filler 65
For a few months he seemed romantic and bought me flowers and chocolates.
Did he buy chocolates and flowers? Y
Filler 66
My neighbor told me it was a long affair that she would rather forget.
Was it a long affair? Y
Filler 67
He saw there was a bond between the mother and her daughter.
Was there a bond between the mother and the daughter? Y
Filler 68
He was nice enough to pick up the coat that was left behind.
Was there a coat left behind? Y
Filler 69
They selected some props for the play including a saddle and a cowboy hat.
Did they select props for the play? Y
Filler 70

We insisted that it was not our fault that his cousin got so angry.

Did someone get angry? Y

Filler 71

They listened to the advice of the teacher who was well known for his wisdom.

Was the teacher well known? Y

Filler 72

They carefully examined the diagram of the elbow and knee joints.

Was the diagram of elbow and knee joints? Y

Filler 73

I thought that was a weird ending for that movie.

Was the ending of the movie weird? Y

Filler 74

We found out yesterday that it was a female puppy that had been lost.

Was a puppy lost? Y

Filler 75

My friend was annoyed that the husband did not allow his wife to go out.

Was the friend annoyed? Y

Filler 76

It seemed to me that the illness would only get worse.

Was the illness getting better? N

Filler 77

The group of friends were sure that the engagement would last a long time.

Will the engagement be short? N

Filler 78

We heard about the river flowing into the town.

Did the river flow past the town? N

Filler 79

I fell asleep and did not hear whether the murder case had been solved.

Did they find out if the murder case was solved? N

Filler 80

When I was not looking he kept trying to take the pencil away from me.

Was he trying to take a wallet? N

Filler 81

I wrote my friend a letter about the summer I spent in Australia.

Did they spend a summer in Australia? N

Filler 82

They made a space for the painting being shipped in from France.

Was the painting being shipped in from Germany? N

Filler 83

None of us dreamed that the recovery would be so fast.

Did they expect the recovery to be fast? N

Filler 84

We stared at the picture of the tongue and all of its taste bulbs.

Was the picture of a face? N

Filler 85

The officials were satisfied with the treaty that had been signed.

Were the officials unhappy? N

Filler 86

My sister took the tasty sauce to our grandmother.

Was the sauce disgusting? N

Filler 87

The basketball game took place in the brand new arena that was built with taxpayer money.

Did the basketball game take place outdoors? N

Filler 88

Bob swallowed a small chicken bone and began to choke during last night's dinner.

Did Bob swallow a chicken bone? Y

Filler 89

The thieves tried to crack the secret code to open the safe.

Did the thieves try to open the safe? Y

Filler 90

The prince became a frog because the witch had put a curse on him.

Did the witch put a curse on the prince? Y

Filler 91 a

Robert was so cruel we thought he was possessed by the devil and avoided him at all costs.

Was Robert cruel? Y

Filler 93

From the beach we could see the shark's fin pass through the water.

Was there a shark in the water? Y

Filler 94

We vacuumed the rug and mopped the floor to help our parents.

Did they vacuum the rug? Y

Filler 95

The accountant examined how the university spent the funds last year.

Did the accountant examine how the university spent its funds? Y

Filler 96

The mother's voice was warm and gentle as she talked to her new baby.

Did the mother talk to the new baby? Y

Filler 97

The annoyed driver kept honking the car horn while he yelled out the window.

Did the driver honk the horn? Y

Filler 98

During these elections we will be choosing a new town mayor and a new district attorney.

Are the elections for the town mayor and district attorney? Y

Filler 99

She took off all her clothes and jumped in the water completely nude in the middle of the night.

Did she jump in the water in the middle of the night? Y

Filler 100

He was so frightened that his lips trembled and his face was deathly pale from the sight of the accident.

Was he calm when he saw the accident? N

Filler 101

I leave a bacon frying for a while in the pan until it becomes crisp.

Did the bacon catch on fire? N

Filler 102

I sliced apples because I was going to bake a pie for my dinner guests that evening.

Were peaches being sliced? N

Filler 103

The child saw the new toy and wanted to play with it right away.

Did the child see a dog? N

Filler 104

He was so embarrassed his face turned bright red and I thought he would cry.

Was he proud? N

Filler 105

He had hung himself from the ceiling with a piece of rope and was discovered the next day.

Did he hang himself from a tree? N

Filler 106

My daughter tried to forge my signature on her report card.

Did the daughter try to forge a check? N

14 1

Filler 107

The groom pulled back the bride's veil to see her blushing face.

Did the groom push the bride? N

Filler 108

There was a lot of dust in the air conditioner's vent which needed to be cleaned.

Was the air conditioner's vent clean? N

Filler 109

I put the flowers in the ceramic vase in the dining room.

Was the vase made of metal? N

Filler 110

Between the two buildings was a long and dark alley filled with garbage cans.

Was the alley filled with stores? N
Filler 111
Mike never offered to pay for anything because he was so cheap and she was very much annoyed.
Did Mike always offer to pay? N
Filler 112
When the winning goal was scored the fans began to cheer and celebrate the victory.
Did the fans cheer and celebrate? Y
Filler 113
The detective became impatient as he looked for a clue at the crime scene.
Did the detective become impatient? Y
Filler 114
At the barbeque I ate a burger and an ear of corn fresh from the nearby farm.
Was the corn from a nearby farm? Y
Filler 115
In the museum we saw the king's gold, royal crown locked behind a glass door.
Was the king's gold crown in the museum? Y
Filler 116
Beth moved to the music very well and wanted to become a dancer when she grew up.
Did Beth want to become a dancer? Y
Filler 117
We talked about the cows and chickens we saw when we visited the farm in New York state.
Was there a farm in New York state? Y
Filler 118
The unexpected storm was not predicted in the forecast that we heard on the radio.
Was the storm unexpected? Y
Filler 119
She wanted to hang the portrait so we went shopping for a frame to put it in.
Did she want to hang a portrait? Y
Filler 120

At the pond we could see a green frog jumping in and out of the water.

Was the frog jumping in and out of the water? Y

Filler 121

Interestingly the stories in the magazine were unknown to her for many years.

Did she know the stories in the magazine? N

Filler 122

Fortunately the drawings in the textbook were much better in this edition.

?Were the drawings worse? N

Filler 123

Accidentally the doors to the office were left unlocked by the cleaning service.

Were the doors to the office locked? N

Filler 124

Regrettably the proposals by the committee were under consideration for a long time.

Were the proposals under consideration? Y

Filler 125

Surprisingly the bags for the purchase were left on the counter by the customer.

Were the bags left by the customer? Y

Filler 126

Remarkably the answers to the question were simpler than we had expected.

Were the answers difficult? N

Filler 127

Obviously the reasons for the test were to make sure the effect was reliable.

Were the reasons reliable? Y

Filler 128

Unbelievably the designs of the study were shown to be problematic in subsequent tests.

Were the designs shown to correct? N

Filler 129

Yesterday the roads to the house were covered with water and mud.

Were the roads dry? N

Filler 130

Terribly the words on the screen were hard to be recognized.

Were the words easy to be recognized? Y

Filler 131

Carefully the causes of the accident were under investigation by the local police.

Were the accident under investigation? Y

Filler 132

Surprisingly the boxes for the toy were found in the backyard.

Were the boxes found in the background? Y

Filler 133

Neatly the illustrations in the manual were done by a well-known artist.

Were the illustrations done by a teacher? N

Filler 134

Unfortunately the addresses on the envelope were not clear to the postman.

Were the addresses clear to the postman? N

Filler 135

Sadly the definitions in the dictionary were not helpful for understanding the word.

Were the definitions helpful for understanding? N

Filler 136

Recently the uniforms were made by factories in China.

Were the uniforms made in China? Y

Filler 137

Loudly the teacher introduced the speaker to everyone in the room.

Did the teacher introduce the speaker to everyone? Y

Filler 138

Everybody in the office felt his kindness towards the new worker.

Did everybody feel his kindness? Y

Filler 139

Politely the mother asked her son not to hurt himself while cooking.

Did the mother ask her son not to hurt himself? N

Filler 140

Unexpectedly the lady bought herself a necklace in the mall.

Did the lady buy herself a watch? N

Filler 141

Sensibly the editor wanted the paper to be further revised by the author.

Did the editor want author to revise the paper? Y

Filler 142

Amazingly the company named Susan the employee of the year this morning.

Was Susan name the employee of the year? Y

Filler 143

307 a Certainly your boss expected you to be there as early as possible.

Did the boss expect you to be there? Y

Filler 144

Reasonably the advisor asked him to talk to his colleagues about it.

Did the advisor ask him to talk his colleagues about it? Y
