# Supplementary material for: Lexical and Grammatical Aspect in On-line Processing of English Past Tense and Progressive Aspect by Mandarin Speakers
Source: Front Psychol. 2021 Jun 10;12:661923. doi: 10.3389/fpsyg.2021.661923 (PMC8222903; doi:10.3389/fpsyg.2021.661923)
Supplement: Supplementary file 3 [file Data_Sheet_2.PDF]

Models and Results of the Statistical Analysis (Linear Mixed Effect Models) of Online Processing English Past Tense and Progressive Aspect

1. Overall

model.11 <- lmer(log(V1) ~ TA*GROUP*TYPE + (1|SUBJ) + (1|ITEM),data= x_01, REML = FALSE)

summary(model.11)

library(lsmeans)

library(emmeans)

lsmeans(model.11,pairwise ~ GROUP * TYPE * TA, adjust = "tukey")

emms1 <- emmeans(model.11,~ GROUP*TYPE*TA)

contrast(emms1,interaction = "pairwise")

afex::mixed(log(V1) ~ GROUP*TYPE*TA + (1|ITEM) + (1|SUBJ),data =x_01,

method = "LRT")

Effects	df	Chisq	p.value
GROUP	2	169.64 ***	<.001
TYPE	2	128.21 ***	<.001
TA	1	1.96	.162
GROUP:TYPE	4	7.02	.135
GROUP:TA	2	21.93 ***	<.001
TYPE:TA	2	77.92 ***	<.001
GROUP:TYPE:TA	4	67.53 ***	<.001

```
Fixed effects:
                        Estimate Std. Error t value
(Intercept)             6.931748   0.018165 381.607
TAPRO                  -0.153595   0.024472  -6.276
GROUPCH_L               0.072761   0.023270   3.127
GROUPNS                -0.184115   0.023292  -7.905
TYPEp                  -0.178376   0.024450  -7.295
TYPEs                   0.098420   0.024650   3.993
TAPRO:GROUPCH_L        -0.071755   0.032972  -2.176
TAPRO:GROUPNS           0.174010   0.033049   5.265
TAPRO:TYPEp             0.250955   0.034638   7.245
TAPRO:TYPEs             0.135861   0.034950   3.887
GROUPCH_L:TYPEp        -0.081567   0.033066  -2.467
GROUPNS:TYPEp           0.134953   0.033034   4.085
GROUPCH_L:TYPEs         0.001052   0.033382   0.032
GROUPNS:TYPEs           0.084455   0.033150   2.548
TAPRO:GROUPCH_L:TYPEp   0.225314   0.046751   4.819
TAPRO:GROUPNS:TYPEp    -0.153951   0.046749  -3.293
TAPRO:GROUPCH_L:TYPEs   0.063741   0.047193   1.351
TAPRO:GROUPNS:TYPEs    -0.107112   0.046971  -2.280
```

2. Past tense

```
model.11 <- lmer(log(V1) ~ GROUP*TYPE + (1|SUBJ) + (1|ITEM),data= x_01, REML =
FALSE)

summary(model.11)

library(lsmeans)

library(emmeans)

lsmeans(model.11,pairwise ~ GROUP * TYPE, adjust = "tukey")

emms1 <- emmeans(model.11,~ GROUP|TYPE)

contrast(emms1,interaction = "pairwise")

afex::mixed(log(V1) ~ GROUP*TYPE + (1|ITEM) + (1|SUBJ),data =x_01,
        method = "LRT")
```

regions	Effects	df	Chisq	p.value
V	GROUP	2	151.57 ***	<.001
	TYPE	2	76.08 ***	<.001
	GROUP:TYPE	4	47.76 ***	<.001
V+1	GROUP	2	151.89 ***	<.001
	TYPE	2	443.89 ***	<.001
	GROUP:TYPE	4	47.08 ***	<.001
V+2	GROUP	2	153.86 ***	<.001
	TYPE	2	535.37 ***	<.001
	GROUP:TYPE	4	62.86 ***	<.001
SF	GROUP	2	162.14 ***	<.001
	TYPE	2	508.44 ***	<.001
	GROUP:TYPE	4	67.37 ***	<.001

Results At the critical region

```
Fixed effects:
                  Estimate Std. Error t value
(Intercept)       6.931894   0.018025 384.562
GROUPCH_L         0.072501   0.022285   3.253
GROUPNS          -0.184037   0.022305  -8.251
TYPEp            -0.178577   0.023835  -7.492
TYPEs             0.098012   0.024024   4.080
GROUPCH_L:TYPEp -0.080888   0.031666  -2.554
GROUPNS:TYPEp     0.132085   0.031650   4.173
GROUPCH_L:TYPEs   0.002034   0.031971   0.064
GROUPNS:TYPEs     0.084733   0.031746   2.669

Correlation of Fixed Effects:
                (Intr) GROUPCH_L GROUPNS TYPEp  TYPEs  GROUPCH_L:TYPEp GROUPNS:TYPEp
GROUPCH_L       -0.617
GROUPNS         -0.616  0.499
TYPEp           -0.657  0.467     0.466
TYPEs           -0.652  0.463     0.462   0.493
GROUPCH_L:TYPEp  0.434 -0.704    -0.351  -0.662 -0.326
GROUPNS:TYPEp    0.434 -0.351    -0.705  -0.662 -0.326  0.498
GROUPCH_L:TYPEs  0.430 -0.697    -0.348  -0.325 -0.662  0.491           0.245
GROUPNS:TYPEs    0.433 -0.350    -0.703  -0.327 -0.667  0.246           0.495
                GROUPCH_L:TYPEs
GROUPCH_L
GROUPNS
TYPEp
TYPEs
GROUPCH_L:TYPEp
GROUPNS:TYPEp
GROUPCH_L:TYPEs
GROUPNS:TYPEs    0.501
>
```

3. Progressive

model.11 <- lmer(log(V1) ~ GROUP*TYPE + (1|ITEM) + (1|SUBJ),data= x_01, REML = FALSE)

summary(model.11)

library(lsmeans)

lsmeans(model.11,pairwise ~ GROUP * TYPE, adjust = "tukey")

library(emmeans)

library(lsmeans)

emms1 <- emmeans(model.11,~ GROUP|TYPE)

contrast(emms1,interaction = "pairwise")

afex::mixed(log(V1) ~ GROUP*TYPE + (1|ITEM) + (1|SUBJ),data =x_01,

method = "LRT")

regions	Effects	df	Chisq	p.value
	GROUP	2	49.16 ***	<.001
V	TYPE	2	69.78 ***	<.001
	GROUP:TYPE	4	27.39 ***	<.001
	GROUP	2	49.21 ***	<.001
V+1	TYPE	2	303.83 ***	<.001
	GROUP:TYPE	4	27.42 ***	<.001
	GROUP	2	47.33 ***	<.001
V+2	TYPE	2	304.42 ***	<.001
	GROUP:TYPE	4	22.97 ***	<.001
	GROUP	2	47.01 ***	<.001
SF	TYPE	2	302.27 ***	<.001
	GROUP:TYPE	4	22.62 ***	<.001

Results At the critical region

```
Fixed effects:
                 Estimate Std. Error t value
(Intercept)      6.777995   0.018928 358.097
GROUPCH-L        0.001145   0.024231   0.047
GROUPNS         -0.009918   0.024323  -0.408
TYPEp            0.075082   0.024825   3.024
TYPEs            0.237754   0.025055   9.489
GROUPCH-L:TYPEp  0.142861   0.034251   4.171
GROUPNS:TYPEp   -0.021617   0.034299  -0.630
GROUPCH-L:TYPEs  0.062914   0.034551   1.821
GROUPNS:TYPEs   -0.025918   0.034484  -0.752

Correlation of Fixed Effects:
                (Intr) GROUPCH-L GROUPNS TYPEp  TYPEs  GROUPCH-L:TYPEp GROUPNS:TYPEp
GROUPCH-L       -0.646
GROUPNS         -0.644  0.503
TYPEp           -0.659  0.493     0.491
TYPEs           -0.653  0.488     0.486   0.498
GROUPCH-L:TYPEp  0.457 -0.707    -0.356  -0.693 -0.345
GROUPNS:TYPEp    0.456 -0.357    -0.709  -0.692 -0.345  0.502
GROUPCH-L:TYPEs  0.453 -0.701    -0.353  -0.345 -0.694  0.496           0.250
GROUPNS:TYPEs    0.454 -0.355    -0.705  -0.346 -0.695  0.251           0.500
                GROUPCH-L:TYPEs
GROUPCH-L
GROUPNS
TYPEp
TYPEs
GROUPCH-L:TYPEp
GROUPNS:TYPEp
GROUPCH-L:TYPEs
GROUPNS:TYPEs    0.504
```

```
> contrast(emms1,interaction = "pairwise")
TYPE = a:
 GROUP_pairwise  estimate     SE   df t.ratio p.value
 (CH-H) - (CH-L) -0.00115 0.0243 2324 -0.047  0.9623
 (CH-H) - NS      0.00992 0.0244 2325  0.407  0.6839
 (CH-L) - NS      0.01106 0.0242 2325  0.456  0.6481

TYPE = p:
 GROUP_pairwise  estimate     SE   df t.ratio p.value
 (CH-H) - (CH-L) -0.14401 0.0242 2324 -5.941  <.0001
 (CH-H) - NS      0.03154 0.0242 2324  1.302  0.1929
 (CH-L) - NS      0.17554 0.0242 2325  7.249  <.0001

TYPE = s:
 GROUP_pairwise  estimate     SE   df t.ratio p.value
 (CH-H) - (CH-L) -0.06406 0.0247 2325 -2.598  0.0094
 (CH-H) - NS      0.03584 0.0245 2325  1.464  0.1433
 (CH-L) - NS      0.09990 0.0244 2325  4.090  <.0001
```
