# Supplementary figures and images for: Lexical and Grammatical Aspect in On-line Processing of English Past Tense and Progressive Aspect by Mandarin Speakers
Source: Front Psychol. 2021 Jun 10;12:661923. doi: 10.3389/fpsyg.2021.661923 (PMC8222903; doi:10.3389/fpsyg.2021.661923)

**Supplementary Figure 1: Mean log frequency of critical verbs in their inflected forms**

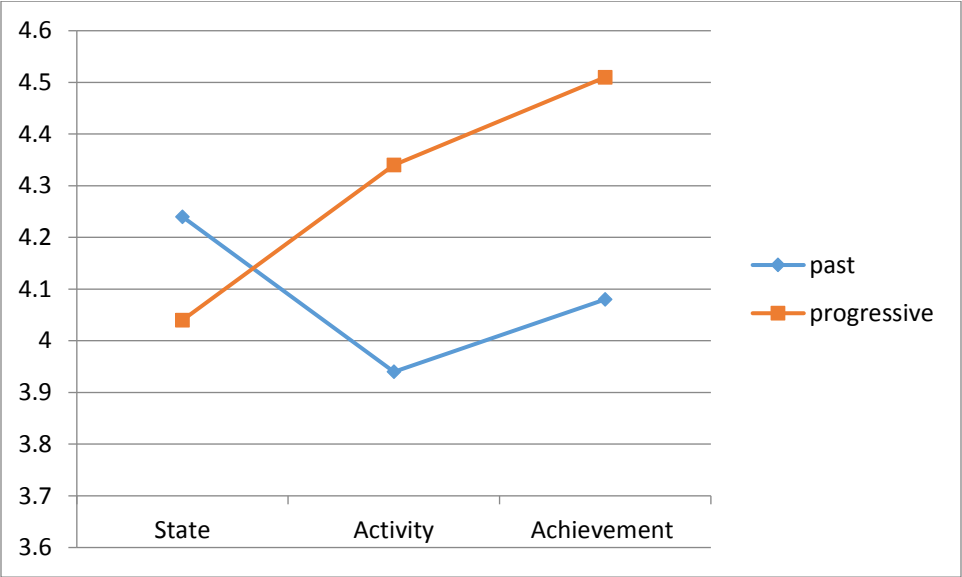

Supplement: Supplementary file 4 [file Image_1.pdf]
